# Chromosome segregation and chromatin integrity in spermatozoa from a t(2;8)(p24;p21)mat carrier: A case-report

**Published:** 2018-09

**Authors:** Catalina García-Vielma, Martha Imelda Dávila-Rodríguez, Elva Irene Cortés-Gutiérrez

**Affiliations:** 1 *Department of Genetics, Centro de Investigación Biomédica Del Noreste (CIBIN), Instituto Mexicano Del Seguro Social (IMSS), Monterrey, Nuevo León, México.*; 2 *Faculty of Biological Sciences, Universidad Autónoma de Nuevo León, UANL, Monterrey, Nuevo León, México.*

**Keywords:** Infertility, Male, Spermatozoa, DNA damage, Chromosome segregation, Translocations, Genetic

## Abstract

**Background:**

Chromosome rearrangements can produce genomic imbalance in gametes which causes a drastic decrease in fertility. Several studies have described the relationship between high levels of DNA damage and chromosomal alterations in the spermatozoa of infertile or subfertile males. However, the nature of this relation is poorly understood. In this study, the meiotic segregation pattern and chromatin integrity were analyzed in the ejaculated spermatozoa of a 46, XY, t(2;8)(p24;p21)mat carrier with normozoospermia and a lack of conception.

**Case:**

A 39-year-old infertile man with a 46, XY, t(2;8)(p24;p21)mat inherited from his mother, was studied. The wife of the proband (30 yrs old) had a normal karyotype and no reproductive problems. The meiotic segregation pattern and aneuploidy of chromosome-8 and chromosome-2 were analyzed by FISH. Sperm DNA damage was evaluated by the Sperm Dispersion Chromatin, alkaline comet assay and DNA breaking detection. Five healthy male donors were included as controls. The frequency of genetically unbalanced spermatozoa was 61.6%. Analysis of the aneuploidy of chromosome-8 and chromosome-Y revealed approximately three and 24 fold increased level respectively in comparison with that of the control group.

**Conclusion:**

We suggest that the accumulation of genetically unbalanced spermatozoa, and increased sperm aneuploidy level is related to male infertility. Interestingly, the case described here has a high level of sperm chromosomal imbalance appears to be linked to sperm DNA fragmentation status. This information could be useful in assisted reproductive techniques.

## Introduction

Infertility affects almost 20% of couples in reproductive age and the male factor being responsible for 30-50% of this infertility. Thus, the individual etiology is important for the treatment. There are germinal chromosomal alterations approximately 10 times more in infertile men than the general population (1). About 10% of genes in the genome are related to spermatogenesis. Balanced translocations as reciprocal translocations and Robertsonian translocations are the most common chromosomal alterations in male infertility. The carriers of these rearrangements have risk of offspring with fetal malformations (2) or repeated abortions due to monosomies or trisomies present in sperm (3). Chromosomal defects may affect the development of male gonads and the urogenital tract. Moreover, abnormalities in germ cell maturation lead to the production of malfunctioning spermatozoa (4). 

High levels of DNA damage and the presence of chromosomal alterations in sperm of males with infertility or subfertile have been described. However, the nature of this DNA damage and their association with chromosome abnormalities is unclear. To date, few systematic reports examining meiotic segregation, aneusomy, and DNA fragmentation analysis in translocation carriers have been conducted (5-7). Understanding male factor infertility requires detailed knowledge of normal sperm development and function. The purpose of this study was to define the meiotic segregation pattern in the ejaculated spermatozoa of a balanced translocation carrier [46, XY, t(2; 8) (p24; p21)mat], as well as the sperm aneuploidy level of chromosome-8 and chromosome-Y. 

In addition, sperm DNA fragmentation status was evaluated using the SCD test, alkaline comet assay, and DNA breakage detection (DBD)-Fluorescent *in situ* hybridization (FISH).

## Case report

**Patient**

A 39-yr-old infertile man with infertility by three recurrent miscarriage, and 46, XY, t(2;8)(p24;p21)mat carrier was studied. Semen analysis revealed normozoospermia. The presence of recent illness, high fever, seminal hyperviscosity or primary hormonal abnormalities was discarded. The patient presented varicocele (8 years ago) treated by varicocelectomy. His wife (30 yr old) had a normal karyotype and no reproductive problems (son born in 1994 with another partner). Recently the couple had treatment of assisted fertilization; unfortunately, we lost contact with the patient. 

For analysis of aneusomies and sperm DNA fragmentation, in healthy male controls, as it was not possible to include controls related to the case five healthy male donors (age range, 25-30 yr) with normozoospermia according to the criteria of the WHO 2010 (8) and normal karyotypes were included as controls. Ejaculated sperm samples were collected after 3-5 days of sexual abstinence. After liquefaction and washing with PBS (pH 7.4; Sigma-Aldrich, St. Louis, MO), sperm samples were fixed with a fresh fixative solution (methanol: acetic acid, 3:1 v/v, -20^o^C) and then stored at -20^o^C until further use. 

**Cytogenetic analysis**

Karyotyping was performed from blood lymphocyte cultures using G-bands by trypsin/Giemsa (GTG) analysis. Fifty metaphases were analyzed (9).

**Slide preparation**

Sperm samples were fixed with 3:1 methanol: acetic acid, spread onto slides and stored in a freezer at -20^o^C until the FISH procedure. The slides were incubated in a decondensation solution (NaOH 1N) for 2 min, rinsed in distilled water, passed through a series of ethanol 70%, 90%, and 100% for 1 min each and then air-dried. Next, the slides were incubated in formamide 70%, 72^o^C for 2 min, ethanol 70%, 90%, and 100% for 1 min each, and then air-dried. Fixed lymphocyte cultures were spread onto slides directly before FISH.

**FISH**

FISH was performed according to the manufacturer’s (Poseidon FISH DNA probe. Kreatech, Diagnostics. Leica Biosystems; Nussloch, Germany) protocol on lymphocytes and sperm cells using a set of fluorescent centromeric and subtelomeric probes. To determine the segregation type and nuclear chromosomal content of each sperm, we used subtelomeric probes of chromosome-2 pter (green) and chromosome-8 pter (red). The hybridization of centromeric probes of chromosome-8 (CEP 8) was marked in red (D8Z1, red), and of chromosome-8 was marked in green (D8Z1, green); results in yellow were used as internal control. 

For analysis of the aneuploidy, we used centromeric probes of chromosome-8 (CEP 8, D8Z1 alfa satellite, red) and chromosome-Y (DYZ1, satellite III orange) (Vysis, Inc., Abbot Laboratories, Downers Grove, IL, USA). Probes were first tested on chromosomes using conventional cytogenetic and FISH techniques. The success of the FISH experiments was 97%. For analysis, 500 sperm cells /individual were assessed using a Zeiss AxioPhot microscope (Carl Zeiss, Göttingen, Germany) equipped with a proper filter set (DAPI/FITC/Red Texas/Triple) and 20× and 100× oil-immersion objectives. 

The images were impressed using an AxioCam MRm Zeiss 16-bit black-and-white charge-coupled device in a 12-bit TIFF format. The same person performed the analysis.

**Sperm DNA fragmentation**

Three methodologies were used to evaluate DNA damage in sperm: SCD test, alkaline comet assay and DBD-FISH. In SCD test, fragmented sperm cells produce a characteristic halo, representing DNA loops after of extraction of nuclear proteins and acid denaturation. Non-fragmented sperm do not halo produce. The SCD test was developed as the Halosperm® kit according to the manufacturer’s (Halotech DNA, Madrid, Spain). 

We analyzed 300 sperm cells per sample under a microscope with objective of 100×. Five categories of halos were considered according to Fernández and colleagues criteria (10); (a) sperm with large halos and (b) medium-sized halos were considered as non-fragmented. Sperm cells with a very-small-sized halo (c) and, sperm cells without a halo (d) were considered as fragmented. Sperm cells without a halo, but weakly or irregularly stained were considered as severe DNA fragmentation or “degraded (e)” (see figures 5A and 5D). Sperm without these characteristics were excluded from the analysis. 

The comet assay methodology was performing according with Singh and colleagues (11). The slides were stained with propidium iodide (1 µg/mL) in Vectashield (Vector Laboratories, Burlingame, CA, USA), and length of the tails (in µm; Mean±SD) of one hundred cells/individual was obtained. DBD-FISH consists in DNA protein depletion and ssDNA formation by alkaline treatment. It allows evidence ruptures in alkaline labile sites and abasic sites. Subsequently these ssDNA are hybridized with specific probes labeled with fluorescence and quantified by image analysis software Image J (http://rsb.info.nih.gov/ij/)

DBD-FISH in sperm was performed according to Fernández and colleagues. When the DNA breaks increase, the alkali produces more single-stranded DNA and more probe hybridizes, increasing levels of fluorescence (12). Samples were analyzed in Zeiss Axiophot fluorescence microscope (Carl Zeiss, Göttingen, Germany) with specifics filters (DAPI, FITC and Texas red). Image J (http://rsb.info.nih.gov/ij/) was used to analyze 50 sperm nuclei by sample.

**Cytogenetic study**

**Figure 1:**
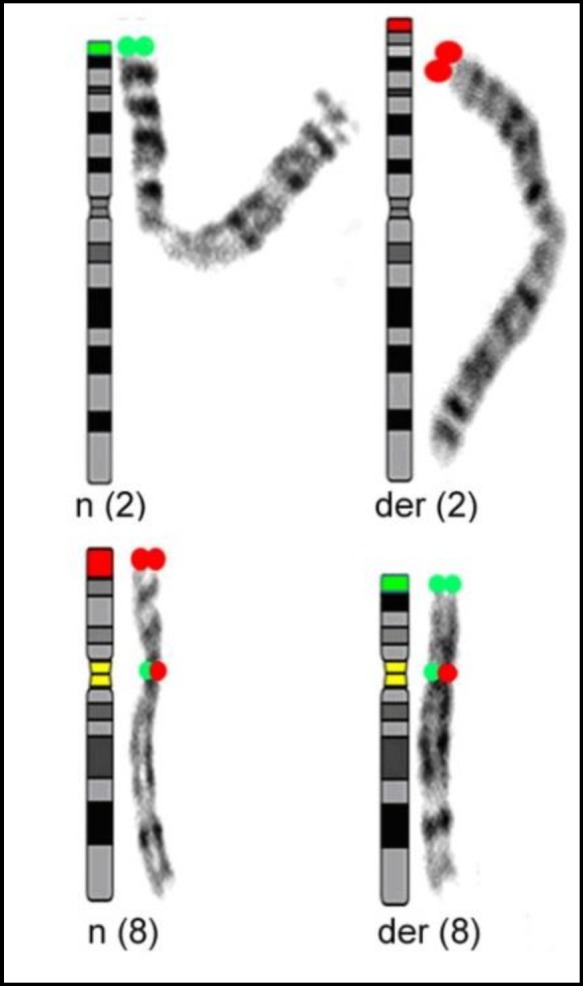
Ideograms of involved chromosomes and FISH labeling scheme for translocation t(2;8)(p24;p21)mat (telomere region of chromosome-2 in green, and of chromosome-8 in red). The centromeric regions of chromosome-8 (red+green=yellow) are internal control. n=normal, der=derivative

Chromosomal analysis of the infertile patient revealed a balanced translocation involving chromosomes-2 and -8; 46, XY, t(2;8)(p24;p21)mat karyotype. Partial karyotypes from GTG banding with ideograms of the chromosomes involved in the balanced translocation are shown in Figure 1. FISH confirmed the presence of this translocation. 

**Segregation pattern**

**Figure 2:**
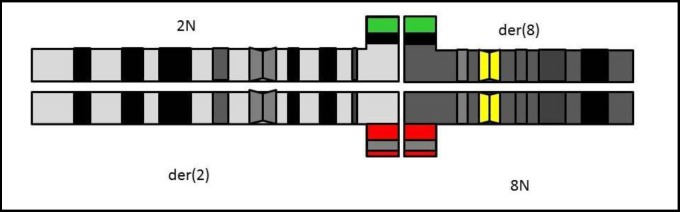
Schematic model of the suggested quadrivalent configuration during the pachytene stage in meiosis I in the 46,XY,t(2;8)(p24;p21)mat carrier. Telomeric region of chromosome-2 (green), and telomeric region of chromosome-8 (red). The centromeric regions of chromosome-8 (yellow) are internal control

**Figure 3:**
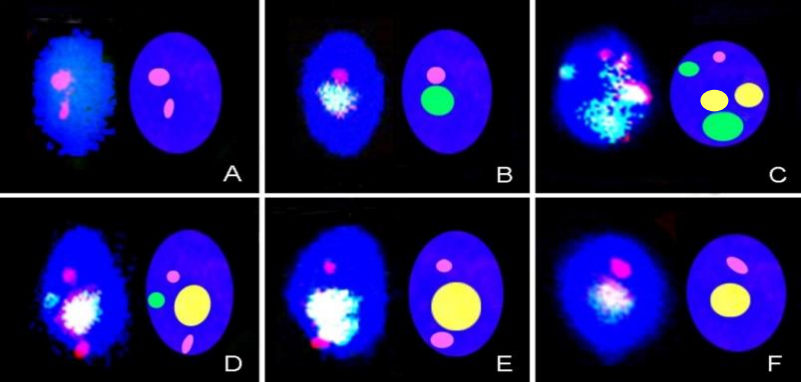
Examples of sperm cell FISH patterns observed in the 46,XY,t(2;8)(p24;p21)mat carrier (telomeric probes; chromosome-2 (green) and chromosome-8 (red). The centromeric regions of chromosome-8 (yellow) are internal control (+). Adyacent 1 [E: 8, der(2)] Adyacent 2 [A: der(2), der(2)] [B: 2, der(2)], 3:1 segregation [C: 2, 8, der(8)] [D: 2, der(2), 8] and F: [8]. Scheme that represents type segregation (right of each cell). Fluorescence microscopy, magnification 1000×.

The meiotic segregation pattern (Figure 2) showed a schematic representation of the theoretically predicted tetravalent structure (pachytene stage of meiosis I) with a chromosome labeling system in the analyzed carrier. An analysis of meiotic segregation by FISH revealed that 61.6% of the sperm cells were genetically unbalanced. The following percentages were found in the different segregations: Alternate: 38.4%, Adyacent 1: 15.6%, Adyacent 2; 12.8%, 3:1: 24.8%, 4:0: 4% and others 4.4% (Table I). Figure 3 shows representative examples of different spermatozoa FISH phenotypes that occurred because of the meiotic segregation patterns in the 46, XY, t(2;8)(p24;p21)mat carrier.

**Sperm aneusomies**

**Figure 4:**
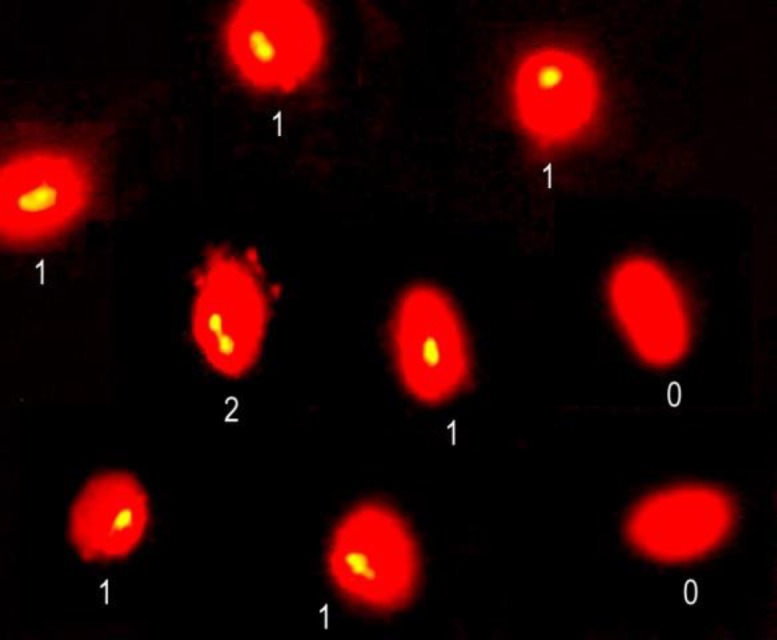
Examples of aneusomic spermatozoa [nullisomic (0), disomic (2)] and normal spermatozoa [monosomic (1)] observed after FISH analysis in the 46,XY,t(2;8)(p24;p21)mat carrier. Centromere-specific FISH probes for chromosome-8 (green) and counterstained with propidium iodide. Fluorescence microscopy, magnification 1000×.

Analysis of the aneusomies of chromosome-8 (nullisomic and disomic) and chromosome-Y(disomic) in the t(2;8) (p24; p21) mat carrier revealed three- and 24-fold increases, respectively, in comparison with that of the control group (Table II). All aneusomy frequencies were significantly higher according to the mean control values (p<0.01). Representative examples of aneusomic spermatozoa are shown in Figure 4.

**Sperm DNA fragmentation**

**Figure 5:**
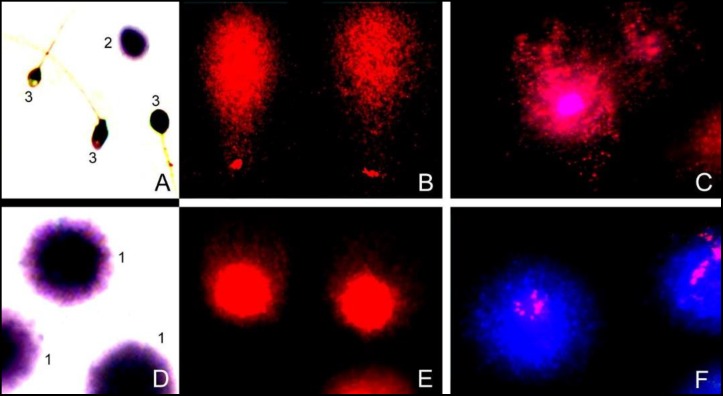
Representative examples of typical staining results for spermatozoa with intact and fragmented DNA in the t(2;8)(p24;p21)mat carrier (top) and controls (bottom) by SCD test stained with Wright 0.28% (A and D), alkaline comet assay (B and E) stained with propidium iodide (1  g/mL), and DBD-FISH using whole probe labeled with biotin and counterstained with DAPI (C and F).

Interesting observations were made during sperm DNA fragmentation evaluation in the t(2;8)(p24;p21)mat carrier. The three tests used (SCD, alkaline comet assay, and DBD-FISH) showed a significant increase compared with those used for the control group (Table III). Interestingly, our patient showed a high percentage of sperm (20%) with severe DNA fragmentation (degraded) in comparison with controls (0.42%). Examples of sperm cells in the t(2;8)(p24;p21)mat carrier and controls after SCD test, the alkaline comet assay, and DBD-FISH are shown in Figure 5.

**Ethical consideration**

All participants were notified of the purpose of the planned research, and written consent was obtained in accordance with to the guidelines of the Research Bioethical Committee, Centro de Investigación Biomédica del Noreste, IMSS. Patient consent was obtained for publication of this article and accompanying images. 

**Statistical analysis**

Comparison of aneusomies (chromosome-8 and chromosome-Y) and DNA fragmentation of spermatozoa between the translocation carrier and control group was performed using the Mann-Whitney test. A value of p<0.05 was considered statistically significant. All analyses were performed using IBM SPSS for Windows 20.0 (Statistical Package for the Social Sciences, IBM Corp., Armonk, NY, USA). 

**Table I:** Meiotic segregation pattern of chromosomes in the sperm cells of the 46,XY, t(2;8)(p24;p21)mat balanced translocation carrier obtained by FISH analysis

**Segregation type**	**Fluorescent phenotypes**	**Sperm (%)**	**Total (%)**
Alternate :			192 (38.40)
2, 8	Green-red/yellow*	112 (22.40)	
de r (2), der (8)	Red-green/yellow*	80 (16)	
Adyacent 1			78 (15.60)
2, der (8)	2 Green – 1 yellow	45 (9)	
8, der (2)	2 Red - 1 yellow	33 (6.60)	
Adyacent 2			64 (12.80)
2, der (2)	1 Green – 1 Red	18 (3.60)	
8, der (8)	1 Green – 1 Red – 2 Yellow	16 (3.20)	
2,2	2 Green	8 (1.60)	
der (2), der (2)	2 Red	9 (1.80)	
der (8), der (8)	2 Green – 2 yellow	7 (1.40)	
8, 8	2 Red – 2 yellow	6 (1.20)	
3:1:			124 (24.80)
2, 8, der (8)	2 Green – 1 Red – 2 yellow	23 (4.60)	
der (2)	1 Red	28 (5.60)	
der (2), der (8), 8	1 Green – 2 Red – 2 yellow	5 (1)	
2	1 Green	6 (1.20)	
2, der (2), 8	1 Green – 2 Red – 1 yellow	7 (1.40)	
der (8)	1 Green – 1 yellow	6 (1.20)	
2, der (2), der (8)	2 Green – 1 red – 1 yellow	24 (4.80)	
8	1 Red – 1 yellow	25 (5)	
4:0 :			20 (4)
2, der (2), der (8), 8	2 Green – 2 Red – 2 yellow	9 (1.80)	
0	Non signal	11 (2.20)	
Others		22 (4.40)	22 (4.40)
Total		500 (100)	500 (100)

* The products of the alternating segregation: green-red/yellow and red-green/yellow were distinguished by the proximity of the red or green signal to the yellow signal of the centromere of chromosome- 8 used as a control mat= Maternal

**Table II:** Aneusomy of chromosome-8 and chromosome-Y detected by FISH in the t(2;8)(p24;p21)mat carrier and men with a normal karyotype

**Aneusomies (%) detected by FISH**		
	**Chromosome -8** a	**Chromosome-Y** b
Patient t(2;8)(p24;p21)mat	1.20	1.70
Control	0.35	0.07
Mann-whitney test	p<0.05	p<0.05

a : Chromosome-8 (nullisomic and disomic) and

b : chromosome-Y (disomic). mat= Maternal

**Table III:** Evaluation of sperm DNA integrity by SCD test, comet assay, and DBD-FISH in the t(2;8)(p24;p21)mat carrier and men with a normal karyotype

	**Sperm DNA Fragmentation**		
	**SCD test (%)**	**Comet assay (** ***um*** **)**	**DBD-FISH (ID)**
Patient	98 *	725.80 ± 117.70 *	69342.17 ± 94383.79*
Control	6.50	90.50 ± 31.29	3970.10 ± 1845.42

ID= Integrated density (segmented area-of-interest x gray-level values)

* Different to control p<0.05

## Discussion

In this study, we evaluated segregation patterns, aneusomies, and DNA fragmentation in the spermatozoa of a balanced translocation carrier t(2;8)(p24;p21)mat with normozoospermia and a lack of conception. FISH technique permit evaluation of a greater number of spermatic nuclei, increasing the sensitive of meiotic segregation patterns studies (13).

To date, few studies have examined meiotic segregates by FISH in the ejaculated spermatozoa of translocation carriers (5-7). Meiotic segregation structure depends primarily on chiasma numbers and asymmetrical multivalents. Analysis of the meiotic segregation of the tetravalent structure by FISH in our patients revealed that 61.6% of the sperm cells were genetically unbalanced, with a prevalence of alternate segregation (38.4%) and Adjacent 3:1 (24.8%). These results are in the range previously reported of unbalanced chromosome complements (69.4-88.3%) (5-7). The two chromosomes evaluated (chromosome-8 and chromosome-Y) had a significantly increased rate of aneusomic gametes compared with the control group (p<0.01). Interchromosomal effect (ICE) is referred for aneuploidies for chromosomes not involved in the translocation. The estimated aneuploidy level (chromosome-Y) in sperm cells of the analyzed t(2;8)(p24;p21)mat carrier indicated the presence of an ICE (14). 

Increased aneuploidy rates have been associated with spermatozoa of males with decreased sperm seminological parameters (OAT: oligo-, astheno-, teratozoospermia). Previous studies indicate an association between ICE presence and oligozoospermia, but no has been observed in cases of astheno- or teratozoospermia (15, 16). The increased aneuploidy level observed in the t(2;8)(p24;p21)mat carrier was not in accordance with previously reported data linking ICE with sperm quality. Although we observed normal parameters for motility concentration, morphology, etc., a high level of sperm DNA fragmentation was detected by the SCD test, comet assay, and DBD-FISH, confirming previously published results (17, 18). Abnormal chromosomal content could be important to its higher sensitivity to exogenous factors of apoptosis and an increase in the DNA fragmentation process (18). According to this hypothesis, spermatozoa with balanced chromosomal abnormalities thus are subjected to DNA fragmentation. 

Interestingly, our patient had a high percentage of sperm with severe DNA fragmentation (degraded). Because he was not exposed at exogenous factors of apoptosis (oxidative stress, heat, radiations, etc.) one explanation for this finding, could be that the presence of immature sperm and abnormalities in chromatin compaction by protamines in patients with chromosomal rearrangements seems to be associated with the presence of the degraded DNA sperm form. DNA fragmentation has been reported in patients with varicocele (19), male infertility and low pregnancy rates (20).

## Conclusion

We suggest the relationship between balanced chromosomal rearrangement, aneusomies increase and DNA fragmentation in spermatozoa from an infertile patient. This information could be useful in assisted reproductive techniques.

## Conflict of interests

The authors declare that there is no conflict of interest regarding this study.
